# Supplementary figures and images for: Identification of the Actin-Binding Region and Binding to Host Plant Apple Actin of Immunodominant Transmembrane Protein of ‘Candidatus Phytoplasma mali’
Source: Int J Mol Sci. 2023 Jan 4;24(2):968. doi: 10.3390/ijms24020968 (PMC9860668; doi:10.3390/ijms24020968)

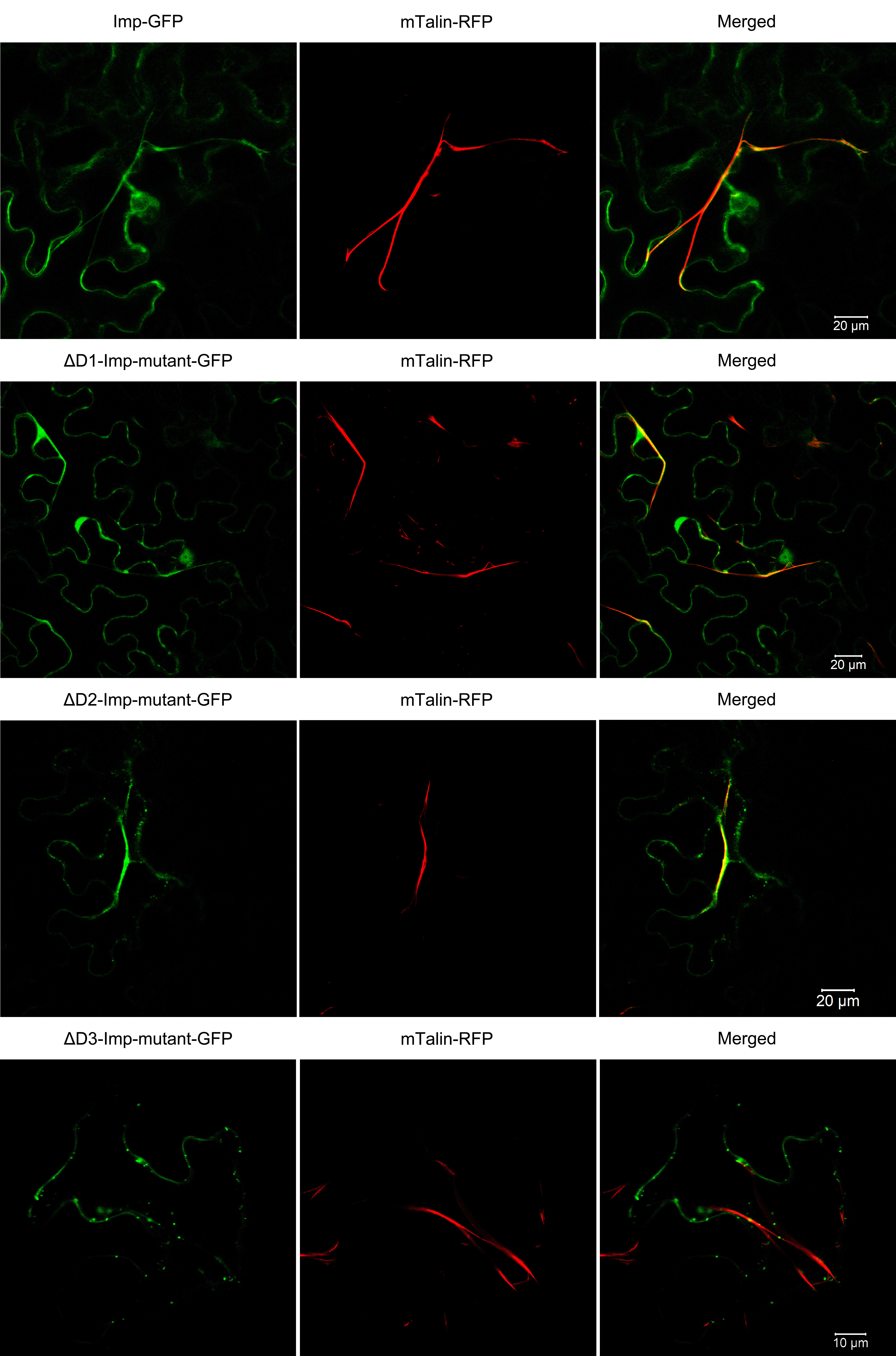

Supplement: Supplementary file 1 [file ijms-24-00968-s001.zip › Figure S1. The magnification of Figure 2.jpg]
